# PHL6/424: Understanding and Controlling Emerging Zoonotic Diseases in an Internet Connected World - A public health veterinarian's approach

**DOI:** 10.2196/jmir.1.suppl1.e87

**Published:** 1999-09-19

**Authors:** S Fagbo

**Affiliations:** ^1^O.V.L, JeddahSaudi Arabia

**Keywords:** Zoonoses, Preventive Medicine, Public Health, Veterinarian

## Abstract

**Introduction:**

The vast progress in communication and information in the last one hundred years has made easy the interaction of people from various parts of the planet. This has benefited the human race in healthcare. The vast leaps in chemotherapy, asepsis and immunology have had a remarkable effect on human life. Technological developments have also led to medical progress. There is however a backlash: the continuously expanding contact have brought to existence hitherto unknown or undocumented diseases - often introduced as emerging diseases. The control of emerging in the year 2000 and beyond may, for the greater part, depend on the optimal use of the Internet, as well as all other forms of advanced telecommunications, integrating a multidisciplinary approach. The new diseases would require at one point or the other the timely and invaluable inputs of the veterinarian and other health care professionals This review is an attempt to examine the vast data available on the internet in respect of zoonoses in order to further understanding and control method: an extensive and continuous search of material on the WWW is attempted. Various search engines were used in searching for relevant key words associated with the topic the discussions and digests of various news groups on the net were (and are still being) analysed in order to glean for current unfolding disease patterns and the reaction of the immediate reaction of the internet medical community to upto date information as well as the integration of the public health veterinarian as and at when required. sites were examined for details on:

- disease reporting
- diagnoses
- prevention/prophylaxis
- public translation of research findings
- integration of various disciplines
- culture

**Results:**

Preliminary results indicate a relative progress in the integration of public health veterinarians as well as other professionals in various areas of emerging disease control. this was however more pronounced in the west. Most news groups are homologous with medical doctors being attached only to medical news groups. Very few veterinarians were found in the medical news groups. There was a direct relation between the increase in new emerging diseases and the ever expanding encroachment of our forests by tourists and farmers. Public translation of research data as well as public awareness seems to be low.

**Discussion:**

Harnessing the vast potentials of the Internet vis a vis the control of emerging diseases will require the harmonisation of a lot of health input/sources on the WWW. Public health curricula should be revised to aid integration of the medical and veterinary profession.

